# Construction of an imaging diagnostic model based on computed tomograph signs for peripheral small cell lung cancer

**DOI:** 10.12669/pjms.41.3.11354

**Published:** 2025-03

**Authors:** Jia Li, Haitao Liu, Cuihong Jiang

**Affiliations:** 1 Jia Li Department of Radiology, Guang’anmen Hospital South Campus, China Academy of Chinese Medical Sciences, Beijing 102618, P.R. China; 2 Haitao Liu Department of Radiology, Guang’anmen Hospital South Campus, China Academy of Chinese Medical Sciences, Beijing 102618, P.R. China; 3 Cuihong Jiang Department of Oncology, Guang’anmen Hospital South Campus, China Academy of Chinese Medical Sciences, Beijing 102618, P.R. China

**Keywords:** Diagnostic model, Non-small cell lung cancer, Peripheral small cell lung cancer

## Abstract

**Objective:**

To construct an imaging diagnostic model for peripheral small cell lung cancer (pSCLC) with a diameter of ≤ 3cm to improve differential diagnostic efficiency.

**Methods:**

As a retrospective study, patients with pathologically confirmed lung cancer with tumor diameter ≤ 3 cm who were treated at the Guang’anmen Hospital South Campus, China Academy of Chinese Medical Sciences from May 2018 to May 2024 were retrospectively selected. All patients underwent computer tomography (CT) imaging. Patients with pSCLC (n=38) were identified first and then matched them to patients with peripheral non-small cell lung cancer (pNSCLC) (n=114) during the same period in a 1:3 ratio. Predictive factors of pSCLC were identified by logistic regression analysis, and a predictive model was constructed.

**Results:**

Logistic regression analysis confirmed that male gender, smooth edges, less spiculation sign, less air bronchogram sign, and lymph node enlargement are independent predictive factors for pSCLC. A predictive model that combines the above five predictive factors has high diagnostic efficacy for pSCLC. The receiver operating characteristic (ROC) analysis results showed the area under the curve AUC of 0.842 (95% confidence interval (CI): 0.759~0.925), with a sensitivity of 84.2% and specificity of 78.1%.

**Conclusions:**

Male sex, smooth edges, less spiculation and air bronchogram signs, and lymph node enlargement identified by the CT scan were shown as independent predictive factors for pSCLC. Combining the above features has a high diagnostic efficacy for pSCLC.

## INTRODUCTION

Lung cancer is a leading cause of cancer-related mortality worldwide and includes two types: non-small cell lung cancer (NSCLC) and small cell lung cancer (SCLC).^1^,^2^ Clinically, lung cancer is classified into peripheral and central based on its location of onset. Peripheral SCLC (pSCLC) is relatively rare, with an incidence rate of 15~30% of the total SCLC cases.^3^,^4^ Compared to NSCLC, pSCLC is associated with a higher degree of malignancy, strong invasiveness, and poor prognosis.^2^–^5^ Therefore, early differential diagnosis of pSCLC is essential to guide the clinical selection of treatment plans and improve the prognosis.^4^–^6^

Chest computed tomography (CT) is currently the most commonly used method for diagnosing lung cancer. Recent studies indicate that clinical data, including tumor markers and recognizable CT features, can clarify the clinical and pathological types of lung cancer.^7^,^8^ pSCLC is less common than central SCLC in clinical practice, but with the prevalence of CT screening for lung cancer, more and more early stage pSCLC have been detected.^9^ However, there are limited imaging studies focusing on pSCLC, and there are also few studies on imaging diagnostic model based on CT signs for pSCLC, especially for pSCLC ≤3 cm in diameter.

This study retrospectively selected clinical data of pSCLC and pNSCLC patients to construct an pSCLC diagnostic model based on the differences between CT features of pSCLC and pNSCLC and analyze its diagnostic efficacy. Our results may provide a reference for diagnosing and evaluating pSCLC.

## METHODS

As a retrospective study, patients with pathologically confirmed lung cancer with tumor diameter ≤ 3 cm who were treated at the Guang’anmen Hospital South Campus, China Academy of Chinese Medical Sciences from May 2018 to May 2024 were retrospectively selected. Patients with pSCLC (n=38) were identified first and then matched them to patients with pNSCLC (n=114) during the same period in a ratio of 1:3.

### Ethical Approval:

The ethics committee of our hospital approved this study with the number 2023-062-KY-01, Dated: July 27^th^ 2023.

### Inclusion criteria:

- Patients with pathology-confirmed lung cancer.^10^,^11^
- Peripheral solid nodules with a diameter of ≤ 3 cm.
- First visit patients with complete CT imaging data.

### Exclusion criteria:

- Patients with non-solid nodules.
- Patients with secondary metastatic cancer or multiple lung cancers.
- Patients with malignant tumors in other systems.
- History of lung disease
- Poor CT image quality.

### Examination method:

All patients underwent chest CT examination. The equipment selected was Siemens 16 slice spiral CT and combined imaging 160-slice spiral CT. The patient was assisted in assuming a prone position and guided to take a deep breath and hold their breath to perform CT scanning. A scan was performed from the sternoclavicular joint to the transverse septum. The layer thickness was set to 1.25 mm, the matrix was 512 × 512, the collimation was 1 mm, the spiral velocity was 0.5 second, the tube current was 240-300 mA, and the tube voltage was 120 kV. *Enhanced scan:* 100 ml of non-ionic iodine contrast agent was injected through the elbow vein via a pressure injector at a rate of 2.5-3 ml/s. The scan was done 90 seconds after injection completion. Image data were reconstructed using standard (B40f) and high-resolution (B70f) algorithms with 2.5-3mm reconstruction intervals.

### Image processing:

Two experienced department physicians reviewed all CT image information, and when there was a disagreement, consensus was reached through consultation. CT images were analyzed using a lung window (window level 600 HU, window width 1500 HU) and a mediastinal window (window level 40 HU, window width 350 HU).

### The following imaging features of the lesion were recorded:

Tumor relative enhancement (E_rel_), size (short axis of lung window, long axis of lung window, short axis of mediastinal window, long axis of mediastinal window), density (enhancement, plain scan), tumor disappearance rate (TDR) [TDR=1- minimum diameter of mediastinal window x maximum diameter of mediastinal window/(minimum diameter of lung window x maximum diameter of lung window), minimum and maximum diameters of lung window measured at the lung window, and minimum and maximum diameters of mediastinal window measured at the mediastinal window], lymph node enlargement (short axis>1 cm), pleural effusion, interstitial pneumonia, peripheral emphysema, pleural invasion, pleural indentation sign, vascular disease Bundle sign, enhancement, cavity sign, bronchial air sign, calcification, notch sign, spiculation sign, lobulation sign, edge, shape, position were recorded.

**Fig.1:**
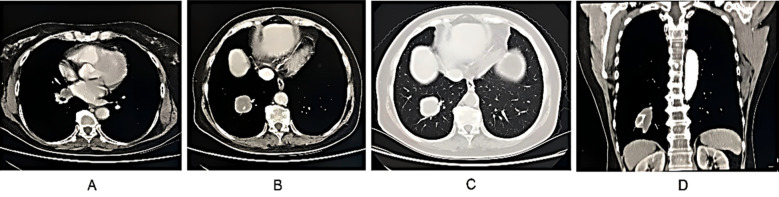
Female patient, 68 years old, with pSCLC in the lower right lung and lymph node metastasis in the right hilum; Imaging features: A-Right lower lung mass, lobulated; B - Smooth edges; C-Vascular passage and stiffness; D-Lymph node enlargement. Puncture pathology: pSCLC with lymph node metastasis.

**Fig.2:**
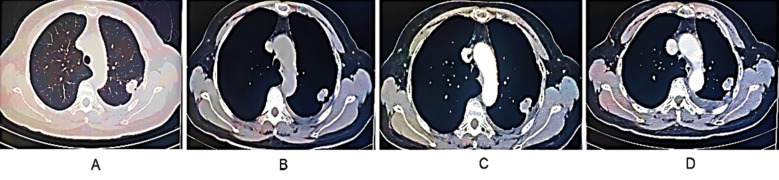
Male patient, 58 years old, with pNSCLC in the upper left lung; Imaging features: A-Left upper lung mass; B-Rough edges; Uneven density; C-After enhancement, the lesion shows moderate to significant uneven enhancement, with indistinct enhancement in the necrotic area; D-There are few slightly larger lymph nodes in the mediastinum, suggesting lung cancer. Pathological result: Adenocarcinoma in the upper left lung.

The TNM staging of the disease was done based on the 8th edition of the AJCC Cancer Staging Manual.^12^ E_rel_=(A_post_-A_pre_)/E_art_; E_art_ is the CT value of the descending aorta at the same level as the lesion; A_pre_ is the attenuation of unenhanced CT of the lesion; A_post_ is the enhanced CT value of the lesion. The region of interest (ROI) was measured on the same axial image. Evaluation criteria for related signs were as follows: pleural invasion with a contact diameter greater than 1/2 of the pleural attachment nodule contact surface; The vascular bundle sign was characterized by an increase in internal blood vessels/convergence of blood vessels; The pleural indentation sign was triangular/linear, originating from nodules and reaching the pleural surface; The notch sign was a V-shaped depression on the surface of the nodule; The air bronchogram sign was characterized by bronchiectasis within the lesion, which can be observed on multiple consecutive levels; Hollow/cavity sign refered to the presence of air like transparent/low attenuation areas larger than 3mm within the nodule; The spiculation sign was characterized by small, spiky protrusions at the edges of nodules; The deep lobulation sign was characterized by a fan-shaped/wavy arrangement at the edge of the lesion, with a chord to chord ratio of ≥ 2/5; Smooth edges indicated that the nodules were clearly separated from the surrounding lung parenchyma by more than 50% Fig.1 & 2.

### Statistical analysis:

The data were processed using SPSS 25.0 software. The Shapiro-Wilk test was used to evaluate the normality of continuous pregnancy data. Normal distribution data were represented as mean ± standard deviation (SD), and an independent sample t-test was used for inter-group comparison. Non-normally distributed data were represented by a median and interquartile range, and the Whitney U test was used for inter-group comparison. The count data were represented by the number of cases and compared using the Chi-square test. Logistic regression model was used to identified the predictive factors for pSCLC. The area under the receiver operating characteristic (ROC) curve (AUC) was calculated, the optimal threshold was determined based on the Youden index, and the specificity and sensitivity of the model were calculated. P<0.05 indicated a statistically significant difference.

## RESULTS

Univariate analysis showed that there were no significant differences in age, history of smoking, disease stage, TDR, lung window short axis, lung window long axis, mediastinal window short axis, mediastinal window long axis size and enhancement type, tumor location, density, calcification, cavity, pleural invasion, and pleural effusion between pSCLC and pNSCLC patients (P>0.05). However, we noted a significant difference in gender distribution, lesion shape, smooth edges, lobulation sign, spiculation, incisions, pleural indentation sign, edge halo sign, air bronchogram sign, peripheral emphysema, and lymph node enlargement between the two groups (P<0.05), as shown in Table-I. Multivariate logistic regression analysis confirmed that male gender and upper edge smoothness on CT, less spiculation sign, less air bronchogram sign, and lymph node enlargement were all independent predictive factors for pSCLC, as shown in Table-II.

**Table-I:** Comparison of the CT signs between pSCLC and pNSCLC.

Item	pSCLC (n=38)	pNSCLC (n=114)	χ^2^/t/Z	P
Sex (Male/Female), n(%)	32 (84.2)/6 (15.8)	69 (60.5)/45 (39.5)	7.171	0.007
Age (years), mean ± SD	61.8±8.5	60.2±9.4	0.966	0.336
History of smoking (Yes), n(%)	29 (76.3)	79 (69.3)	0.682	0.409
Disease Staging, n(%)			5.206	0.157
I	16 (42.1)	61 (53.5)		
II	7 (18.4)	26 (22.8)		
III	13 (34.2)	26 (22.8)		
IV	2 (5.3)	1 (0.9)		
Tumor size (mm), median (range)				
Tumor disappearance rate	0.25 (0.21-0.32)	0.25 (0.18-0.41)	-0.03	0.976
Short-axis of lung window	15 (14-21)	16.5 (15-22)	-1.486	0.137
Long-axis of lung window	24 (21-27)	23 (16-26)	-1.565	0.118
Short-axis of mediastinal window	15 (13-18)	15 (14-20)	-0.74	0.459
Long-axis of mediastinal window	20.5 (18-23)	19 (16-23)	-1.307	0.191
Shape (circular/polygonal), n (%)	24 (63.2)/14 (36.8)	62 (54.4)/52 (45.6)	12.729	0.004
Edge (smooth/rough), n (%)	30 (78.9)/8 (21.1)	44 (38.6)/70 (61.4)	18.574	<0.001
Lobulation sign (Yes), n (%)	24 (63.2)	65 (57.0)	0.443	0.506
Spiculation sign (yes), n (%)	4 (10.5)	43 (37.7)	9.866	0.002
Notch (Yes), n (%)	26 (68.4)	65 (57.0)	1.543	0.214
Homogenous enhancement (Yes), n (%)	24 (63.2)	79 (69.3)	0.492	0.483
Vessel convergence sign (yes), n (%)	14 (36.84)	44 (38.6)	0.037	0.847
Pleural indentation sign (yes), n (%)	12 (31.6)	53 (46.5)	2.589	0.108
Tumor location (left/right), n (%)	22 (57.9)/16 (42.1)	69 (60.5)/45 (39.5)	0.082	0.774
Calcification (Yes), n (%)	5 (13.2)	10 (8.8)	0.616	0.432
Air bronchogram sign (yes), n (%)	3 (7.9)	34 (29.8)	7.442	0.006
Cavity (Yes), n (%)	5 (13.2)	32 (28.1)	3.441	0.064
Pleural invasion (yes), n (%)	4 (10.5)	28 (24.6)	3.378	0.066
Peripheral emphysema (yes), n (%)	3 (7.9)	11 (9.6)	0.105	0.745
Pleural effusion (yes), n (%)	3 (7.9)	15 (13.2)	0.756	0.385
Lymph node enlargement (yes), n (%)	13 (34.2)	14 (12.3)	9.383	0.002

**Table-II:** Multivariate Logistic Analysis of pSCLS and NSCLS.

Variable	B	S.E.	Waldχ^2^	P	OR	95% CI
Male	1.132	0.545	4.313	0.038	3.101	1.066~3.101
Smooth edges	1.741	0.482	13.048	<0.001	5.703	2.217~14.667
Less spiculation sign	1.269	0.614	4.277	0.039	3.558	1.069~11.844
Less air bronchogram sign	1.477	0.703	4.418	0.036	4.380	1.105~17.362
Lymph node enlargement	1.117	0.54	4.285	0.038	3.056	1.061~8.799
Constant	-5.327	1.01	27.832	<0.001	0.005	

**Fig.3:**
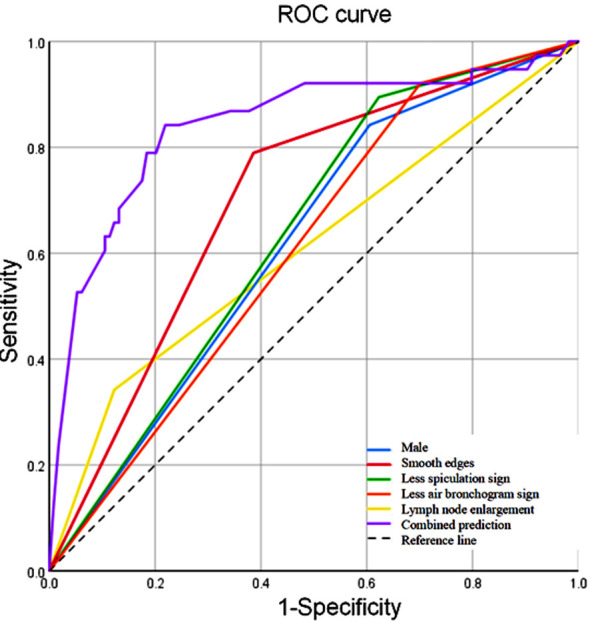
ROC curve of the diagnostic efficacy of the combined model and independent predictive factors for pSCLC.

A prediction model was constructed based on the above five independent predictive factors above. The results showed that combining all these five predictive factors, the ROC AUC of the prediction model was 0.842 (95% CI: 0.759~0.925), with a sensitivity of 84.2% and a specificity of 78.1%, which demonstrated high predictive performance. Table-III and Fig.3.

**Table-III:** Diagnostic efficacy of the combined model and independent predictive factors for pSCLC.

Constant	AUC (95%CI)	Sensitivity (%)	Specificity (%)	P
Male	0.618 (0.521~0.716)	84.2	39.5	0.029
Smooth edges	0.702 (0.609~0.795)	78.9	61.4	<0.001
Less spiculation sign	0.636 (0.542~0.730)	89.5	37.7	0.012
Less air bronchogram sign	0.610 (0.514~0.706)	92.1	29.8	0.043
Lymph node enlargement	0.610 (0.500~0.720)	34.2	87.7	0.043
Combined prediction	0.842 (0.759~0.925)	84.2	78.1	<0.001

## DISCUSSION

Our results showed that pSCLC was more common in male patients and was associated with CT signs that differ from pNSCLC. Male sex, smooth edges, less spiculation sign, less air bronchogram sign, and lymph node enlargement were all identified as independent predictive factors for pSCLC. The model combining the above features had an extremely high diagnostic performance for pSCLC.

Previous studies have identified male gender as an independent risk factor for lung cancer.^11^,^12^ In our study, male patients accounted for 84.2% (32/38) of pSCLC patients. Zhang L et al.^13^ also found that among pSCLC patients, the proportion of males was as high as 88.4% (38/43). It is plausible that this trend relates to the proportion of male smokers, which is related to their lifestyle. Moreover, males are more likely to be exposed to industrial pollutants, which may also lead to the occurrence of lung cancer.^11^–^13^ Studies have shown that smoking is an independent predictor of SCLC.^14^,^15^ Thomas A et al.^15^ also revealed that the characteristics of non-smoking SCLC are lower tumor mutation burden, lower TP53 mutation frequency, and lack of mutation features associated with tobacco exposure. In this study, the smoking rate of pSCLC patients was 76.3%, and the smoking rate of pNSCLC patients was 69.3%, but there was no significant difference between the two. We may speculate that the lack of significant difference may be related to the sample size selection bias. This study found that the proportion of pNSCLC patients with spiculation (37.7%) was significantly higher than that of SCLC patients (10.5%), consistent with previous studies.^16^,^17^ A study by Zhang T et al.^16^ has shown that pSCLC tumor cells usually have a uniform morphology, small cell volume, mainly in the shape of oats or lymphocytes, smooth boundaries, and extensive necrosis inside. Smooth edges are a characteristic feature of pSCLC. In this study, 78.9% of pSCLC patients had smooth edges, significantly higher than pNSCLC (7.86%). As demonstrated by the previous research, CT examination shows that the density of pSCLC is relatively uniform, with smooth edges and circular leaf-like structures.^18^,^19^ Air bronchogram sign is a characteristic of NSCLC.^20^ Air bronchogram refers to the airway exposed through opaque lungs. The airway presents as an inflated, transparent tubular structure, highlighted by the opacity of the surrounding alveoli.^20^,^21^ This study found that the proportion of air bronchogram signs in pSCLC (7.9%) was significantly lower than that in pNSCLC patients (29.8%), and it was an independent predictor of pSCLC. This is consistent with the research of Ma J et al.^17^

Currently, the standard for CT diagnosis of lymph node metastasis is mediastinal or hilar lymph node enlargement with a short diameter ≥ 1 cm.^22^ SCLC has a high degree of malignancy and is highly prone to lymph node metastasis. In agreement with previous research, our results showed that CT lymph node enlargement is an independent predictor of pSCLC.^22^,^23^ This study also presented a predictive model that combined five independent predictors of pSCLC for the differential diagnosis of pSCLC and pNSCLC. The constructed model had a high diagnostic efficacy for pSCLC, with an AUC of 0.842 (95% CI: 0.759~0.925), sensitivity of 84.2%, and specificity of 78.1%. Wong CW et al.^24^ also confirmed that constructing a diagnostic model based on CT imaging and clinical features can differentiate SCLC and NSCLC. Such a model may guide the development or adjustment of intervention plans in clinical practice and is also of great significance for improving targeted and effective treatment.

Future studies should attempt to develop models with additional factors to identify SCLC risk populations better. Wang C et al.^25^ found that higher fruit consumption is negatively correlated with the risk of lung cancer in current and former smokers. At the same time, vegetable intake is significantly associated with reducing the risk of lung cancer in current smokers. These findings may have significant public health implications for preventing lung cancer through dietary interventions. Imaging omics is currently a hot topic in medical imaging research and allows non-invasively obtaining high-throughput imaging omics features of patients from standard medical images. Some studies have shown that this approach can more accurately identify pSCLC and NSCLC.^26^,^27^ The prediction model constructed in this study has the advantages of being simple and easy to implement, with high diagnostic performance, and can provide some reference for the recognition of pSCLC.

### Limitations:

Firstly, due to the small proportion of pSCLC patients and the retrospective single-center nature of the study, the sample size was small, with a possible selection bias. Secondly, we only collected limited clinical data from lung cancer patients. Future studies should aim to include additional clinical data, such as family history and dietary habits, as well as blood biochemical test results and tumor markers. Thirdly, due to the limited number of patients included in this study and the limited number of variables used for model building, the repeatability and robustness of the model need to be validated in prospective multicenter studies with larger datasets.

## CONCLUSION

A predictive model based on CT signs can effectively distinguish pSCLC from pNSCLC. This model may provide a reference basis for disease diagnosis and evaluation and ensure timely targeted interventions.

### Authors’ contributions:

**JL:** Concept and study design, literature search, manuscript writing and revalidation.

**HL** and **CJ:** Data collection, data analysis, interpretation and critical review.

All authors have read, approved the final manuscript and are responsible for the integrity of the study.
